# An Eight-Year Evaluation of a Student-Led Research Pathway: Scholarly Engagement and Residency Match Outcomes in Undergraduate Medical Education

**DOI:** 10.7759/cureus.114533

**Published:** 2026-08-14

**Authors:** Margot Richards, Emma Kane, Rebecca Kann, David Leigh, Mary Petzke

**Affiliations:** 1 School of Medicine, New York Medical College, New York City, USA; 2 Medical Student Research, New York Medical College, New York City, USA

**Keywords:** area of concentration, competitive specialties, medical education, research seminars, residency match, surgical subspecialties

## Abstract

Introduction

Research productivity has become increasingly important for residency applicants, yet many medical students lack structured opportunities to develop research skills. This study evaluated an eight-year, largely student-led research pathway designed to promote scholarly engagement, research training, and residency preparedness among undergraduate medical students.

Methods

We conducted a retrospective evaluation of a student-led research program at a single Liaison Committee on Medical Education (LCME)-accredited US medical school from 2018 to 2025. Program participation was assessed through attendance at research seminars and completion of the Biomedical Research Area of Concentration (AoC). During the 2025-2026 academic year, participants completed surveys evaluating motivations for participation and perceived educational benefits. Residency match outcomes were also examined. Seminar attendance was compared across seven academic-year cohorts. A Kruskal-Wallis H test was used to compare median attendance across years. Trends in Biomedical Research AoC completion and total attendance over time were assessed with a linear least-squares fit, with strength of association quantified by the Pearson correlation coefficient. A Welch’s t-test was used to compare AoC completion rate in competitive and less competitive specialities, and effect size was quantified using Cohen’s d.

Results

A total of 118 research seminars were conducted during the study period. Seminar participation remained robust across eight academic years, with median attendance peaking during the 2020-2021 academic year (p<0.05). Between 2020 and 2025, 193 students completed the Biomedical Research AoC, with annual completion increasing significantly over time (p<0.05). Students matching into competitive residency specialities demonstrated higher rates of AoC completion than those entering less competitive specialities (31.96% vs. 16.13%, p=4.62 × 10⁻⁵). Survey respondents most commonly reported attending seminars to fulfil AoC requirements, presenting research to strengthen their residency applications, and improving presentation skills as the primary educational benefit.

Conclusions

A longitudinal, student-led research pathway was associated with sustained student participation, increasing engagement in structured scholarly activities, and higher participation among students entering competitive residency specialities. These findings suggest that student-led research initiatives may represent a scalable approach to expanding research training and supporting scholarly development within undergraduate medical education.

## Introduction

Medical student research is undoubtedly an integral part of matching into residency programs. A 2020 study found that osteopathic and allopathic medical student applicants who matched into residency programs had a significantly greater number of research accomplishments, including abstracts, presentations, and publications, than applicants who did not match [1]. The importance of research experience in residency applications may be even greater today, given recent changes in medical education and licensing examinations, including the transition of the United States Medical Licensing Examination (USMLE) Step 1 to a pass/fail (P/F) format in 2022 and the increasing adoption of pass/fail grading systems in preclinical medical school curricula.

These changes have reduced the number of factors that residency programs can utilize to compare program applicants. Thus, emphasis has shifted to other metrics of medical student performance, such as research engagement and USMLE Step 2 scores [2]. In response to USMLE Step 1 becoming P/F, students applying to all specialities have reported feeling added pressures to participate in research earlier in medical school, and this pressure to publish is even stronger for those applying to highly competitive subspecialties [3]. One study in 2023 found that having greater than three published papers before beginning one’s intern year was a predictor for matching into a top-25 residency program for neurosurgery applicants [4]. In a survey of plastic surgery residency applicants, participants reported believing that dedicated research time prior to residency would become more important with the change to USMLE Step 1 scoring [2]. Finally, the results of a questionnaire sent to vascular surgery program directors showed that research was an important factor in selecting which applicants to interview [5].

With the path to residency becoming increasingly challenging to navigate, research assistance from peers and mentors during medical school becomes essential for many students. This study investigates the impact of a largely student-led research program at an allopathic LCME-accredited US medical school on multiple aspects of the medical student research experience, including honing of research presentation skills, assistance in identifying research opportunities, and match outcomes. 

This work has been previously presented at the American Medical Association’s Annual Poster Showcase in June 2026 as well as the American Medical Association’s Research Challenge in October 2025.

## Materials and methods

Description of the program

Areas of concentration (AoCs) are a feature of many medical school programs and often act as an adjunct to the academic curriculum [6]. AoCs go by various names at different institutions, but the premise remains largely consistent. AoCs function similarly to an undergraduate minor and generally require completion of additional coursework, projects, or experiences [7]. Some examples of common AoCs include: public health, medical education, health policy, quality improvement, and research [8]. At the institution described in this study, the most popular AoC amongst students is the Biomedical Research track. This AoC was introduced in 2018, prior to the institution and USMLE Step 1 becoming P/F. Requirements for successful completion of the Biomedical Research AoC include: presentation or publication of research, completion of a research methodologies course, devoted research time in the form of an elective, presentation at the annual medical school research fair, and attendance at a minimum of sixteen student-led research seminars. Those who complete the Biomedical Research AoC are awarded with distinction in research at the time of graduation. This study will largely focus on the student-perceived benefits of the research seminars as they are peer-led, interactive, and could serve as an additional component of research AoCs at many institutions. The research seminars are open to all students, and many attendees participate for reasons other than obtaining credit towards the AoC.

The research seminar series is organized and led by a committee of eight medical students. Two students per academic class are elected to the committee during their first year of medical school. This medical student research committee additionally functions as a liaison between students and faculty for the Biomedical Research AoC. In the student-led seminars, selected peers deliver an oral research presentation much like that given in a conference setting. Seminars are held on Zoom, typically during lunch hour, and run for approximately 45 minutes. Typically, two presenters are featured per seminar, and their presentations are connected by a theme. This theme is often a medical speciality (e.g., orthopedic surgery), an area of interest (e.g., preventative medicine), or a specific methodology of study (e.g., quality improvement). Medical students do not have to be committed to the AoC in order to present or attend. The medical student research committee tracks student attendance at each seminar, and this data is utilized to assess for completion of the Biomedical Research AoC at the time of graduation.

Inclusion and exclusion criteria

We conducted a retrospective observational study using previously collected institutional data. For the retrospective analysis, all medical students enrolled in the medical school who participated in the AoC from the 2018-2019 to 2024-2025 academic year were included. Individuals did not have to complete the AoC in order to participate in the research seminars. Individuals who attended the seminars but were not members of the medical school were excluded from attendance counts (e.g., faculty members or participants from other programs). For the fall 2025 semester, motivations for attending the seminars were evaluated from survey responses. 

AoC completion was determined using records maintained by the Office of the Dean of Research. To successfully complete the AoC, students were required to fulfil all program requirements, including attending 16 research seminars, presenting at the annual research forum, publishing scholarly work, and completing the required research methodologies course. Program records were used to evaluate AoC completion and attendance at peer-led research seminars.

For the survey component, two surveys were administered: one to medical students who attended peer-led research seminars during the study period and another to medical students who served as peer-led research seminar presenters. Individuals who attended the seminars but were not medical students (e.g., faculty members or participants from other programs) were excluded from the survey analyses. No surveys were excluded on the basis of incomplete responses. Rather, omitted responses were noted in the response breakdown. The presenter survey was run throughout the academic year, including the spring 2026 semester due to a smaller sample size, while the attendee survey was run exclusively through the fall 2025 semester. 

During the study duration, 193 students completed the AoC, 534 total survey responses were received, and 19 unique presenter survey responses were received. Both surveys were created by the authors (see appendices). This study was approved by our medical college’s institutional review board (New York Medical College Committee for Protection of Human Subjects General Medical and Behavioral Committee with an approval number 24287).

Survey distribution 

In order to gain insight into student motivations for attending the research seminars, attendees were surveyed during the fall of 2025. In order to be marked present for the seminar, attendees were required to answer four questions via a Google Form regarding their participation, including their class year, their primary reason for attending, the speciality they planned to pursue, and if they believed attending the seminar could help them identify research opportunities. Seminar attendance data were obtained and compiled in Google Sheets. Seminar data and metadata, including total attendance, seminar theme, class year of attendees, and seminar date, were also reviewed and compiled. For analysis, seminars were grouped by theme and average attendance and number of seminars were calculated for each theme.

In order to determine motivations for presenting at the seminar and to understand how and what type of medical students benefit from presenting at peer-led research seminars, presenters at the research seminar series were additionally surveyed during the 2025-2026 academic year via an optional post-presentation questionnaire via Google Form. Presenters were asked about their motivations for presenting, how they felt they benefited from presenting, how comfortable they felt answering questions from students and faculty, how many times they had presented before, and what medical speciality they planned to pursue.

AoC completion 

To gauge the impact of the Biomedical Research AoC program as a whole, annual student participant data was provided by the institution’s Office of Undergraduate Medical Education. Data included the number of applicants to each medical speciality and the number of those applicants who completed the Biomedical Research AoC from 2022-2025. Match data for competitive and less-competitive specialities were analysed separately. A competitive speciality was defined as a speciality having an average USMLE Step 1 score ≥ 240 for matched applicants before the scoring change (dermatology, diagnostic radiology, interventional radiology, neurosurgery, ophthalmology, orthopedic surgery, otolaryngology, plastic surgery, urology, vascular surgery) [9].

Statistical analysis 

All statistical analyses were performed in Python (v3.12.12) using SciPy (v1.16.3), NumPy (v2.0.2), and pandas (v2.2.2) in Google Colab; post-hoc comparisons used scikit-posthocs (v0.13.0) [10-12]. Figures were generated with Matplotlib (v3.10.0) and Seaborn (v0.13.2) [13,14].

Seminar attendance was compared across seven academic-year cohorts (2018-2019 through 2024-2025). Because visual inspection of box plots and histograms showed non-normal, heteroscedastic distributions within cohorts, a Kruskal-Wallis H test was used to compare median attendance across years (H(6)=59.31, p<0.001). Given this significant omnibus result, pairwise comparisons were conducted with Dunn's post-hoc test and Bonferroni correction (scikit-posthocs, posthoc_dunn), α=0.05. Six of 21 pairwise comparisons reached significance: 2018-2019 vs 2020-2021 (p=8.4×10⁻⁶), 2019-2020 vs 2020-2021 (p=1.3×10⁻⁴), 2020-2021 vs 2022-2023 (p=1.3×10⁻⁵), 2020-2021 vs 2023-2024 (p=5.9×10⁻¹⁰), 2021-2022 vs 2023-2024 (p=1.7×10⁻³), and 2023-2024 vs 2024-2025 (p=1.3×10⁻³).

Trends in Biomedical Research AoC completion (2020-2025) and total attendance over time were assessed with a linear least-squares fit (NumPy polyfit), with strength of association quantified by the Pearson correlation coefficient (SciPy pearsonr). AoC completion showed a strong positive linear trend (r=0.95); total attendance over time showed no meaningful trend (r=−0.08).

AoC completion rate (the percentage of matched applicants in a speciality who completed the AoC) was compared between competitive and less-competitive specialities for each year from 2022-2025 (n=4 years per group). Because the two groups showed unequal variance in completion percentage, a Welch’s t-test was used, and effect size was quantified using Cohen’s d. Significance for all tests was set at p<0.05.

## Results

**Figure 1:**
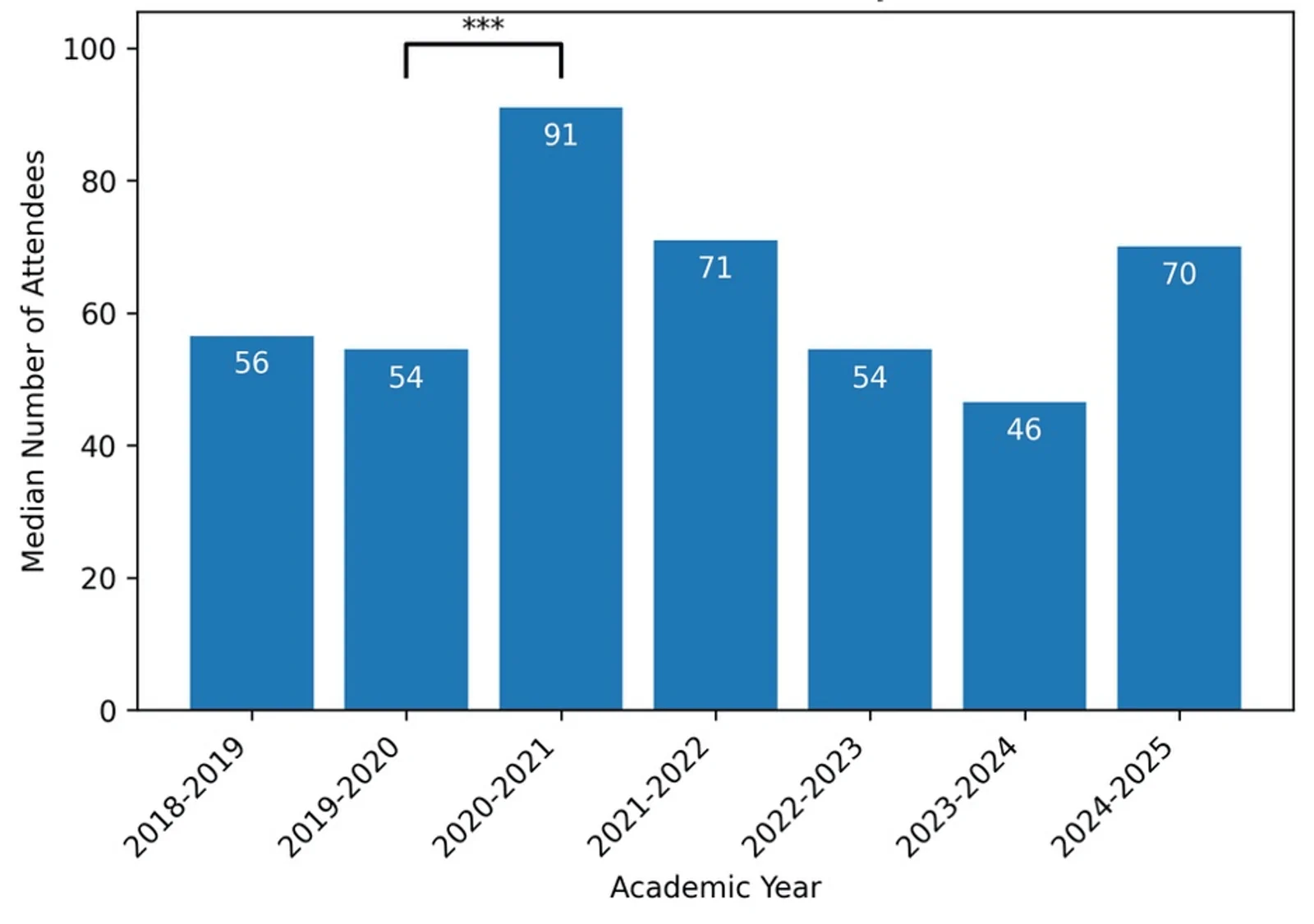
Median Attendance of Attendees by Academic Year. Average number of students attending each seminar by academic year. There was a significant increase in attendees in the year 2020-2021. Dunn’s post hoc test with Bonferroni correction p<0.0005***.

Between academic years 2018-2019 and 2024-2025, 118 research seminars were held with an average attendance of 66.3 students. Median attendance peaked in 2020-2021, which was significantly higher than other years (p<0.05) (Figure 1).

**Figure 2:**
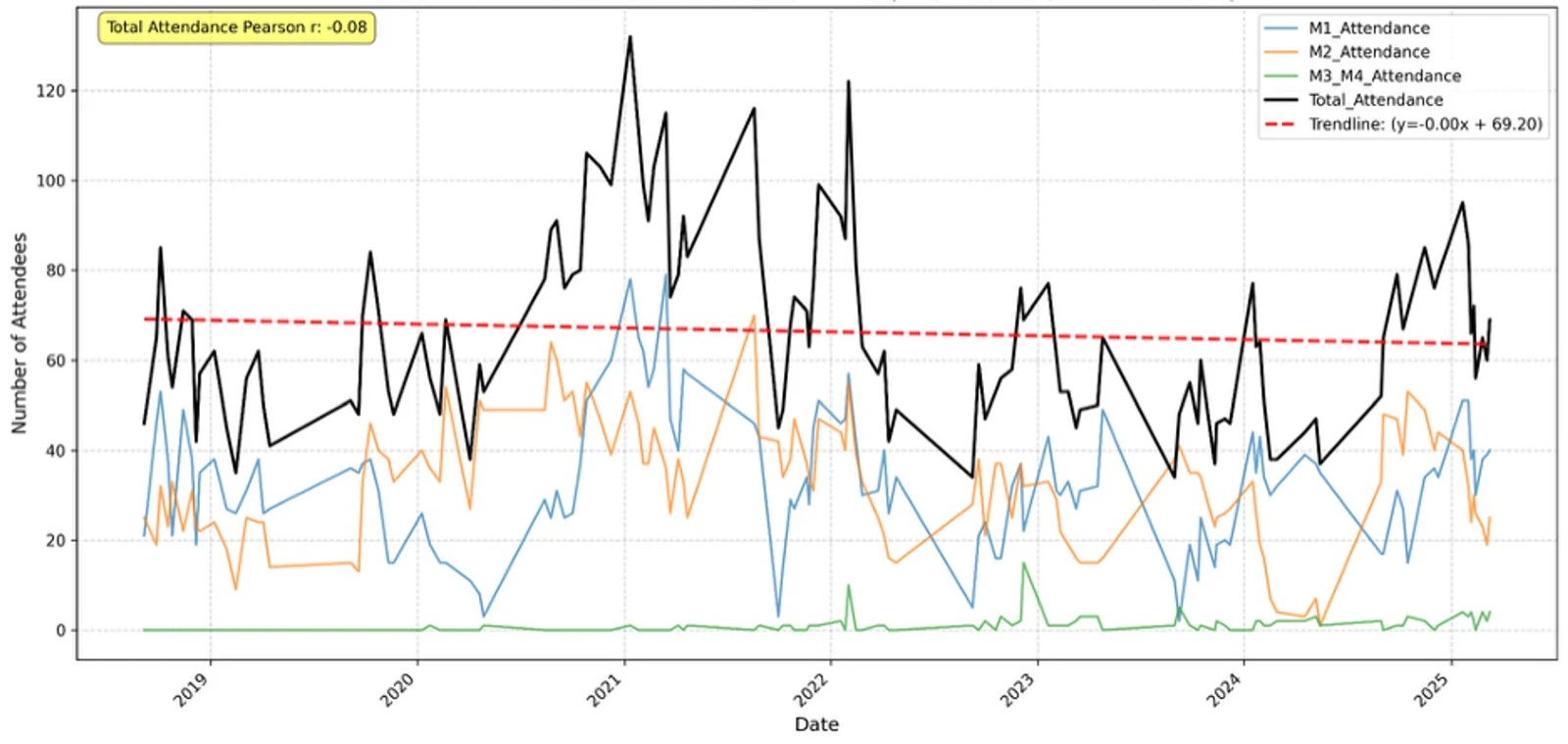
Seminar Attendance Trends Over Time Attendance at the research seminars over time. Black shows total attendance at each seminar. Blue shows the number of first-year medical students attending (M1), and orange shows the number of second year medical students attending (M2). Green shows the number of third and fourth year medical students attending (M3/M4).

First- and second-year medical students were more likely to attend the seminars than third- and fourth-year students (Figure 2).

**Figure 3:**
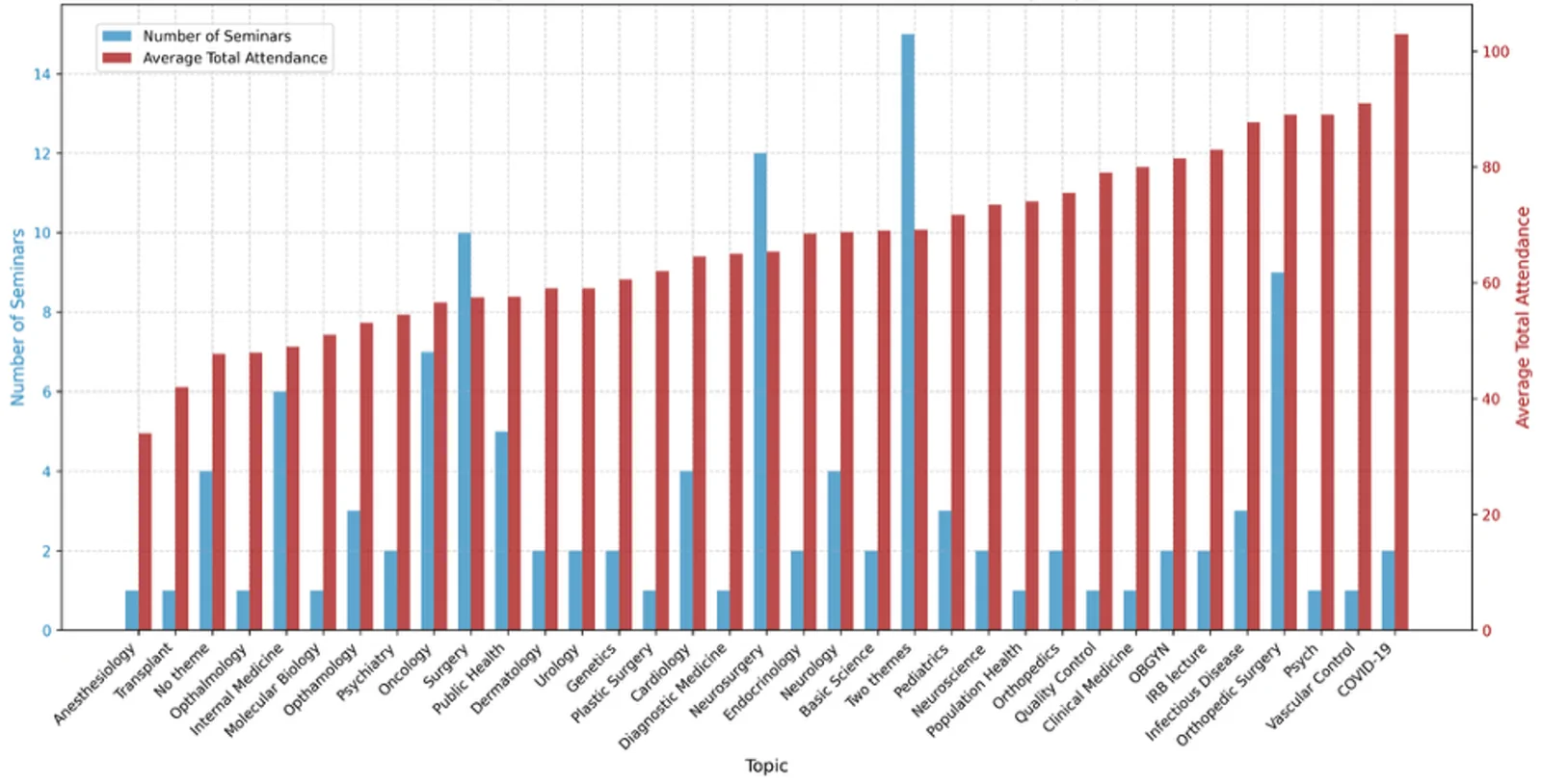
Average Total Attendance vs. Number of Seminars by Topic Average attendance and number of seminars based on seminar themes. The left axis and blue bars show the number of seminars that occurred between September 2018 and March 2025. Graph is in increasing order based on the number of seminars for each theme. Right axis and red bars indicate the average attendance based on each theme. Two themes indicate there were multiple themes listed on the seminar flyer. No theme indicates no specific theme was indicated for the seminar.

The most frequent seminar themes were neurosurgery (12), orthopedic surgery (11), and surgery (10). COVID-19-themed sessions drew the highest average attendance (103), whereas anesthesiology drew the lowest (34) (Figure 3).

**Figure 4:**
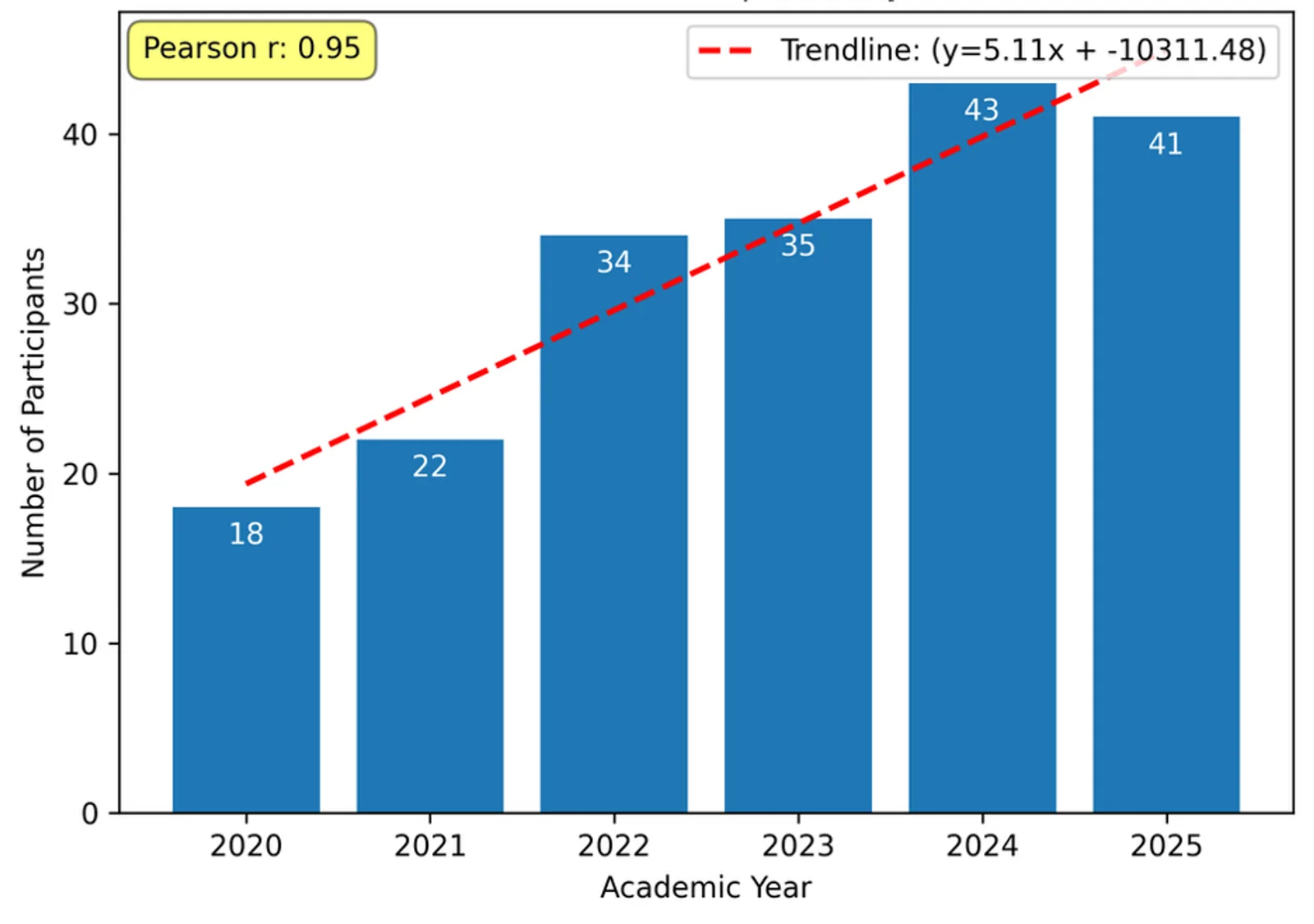
Area of Concentration Participation by Academic Year Number of students completing the Biomedical Area of Concentration (AoC) by year of graduation. There was a positive linear trend in the number of students completing the AoC from 2020 to 2025 (r=0.95, p=0.004). Pearson r of 0.95 indicates there is a strong positive correlation between academic year and number of participants and accounts for a large proportion of the variation in attendance.

From 2020 to 2025, 193 students completed the Biomedical Research AoC with an average of 38.6 students per year. There was a statistically significant positive linear trend in the number of students completing the AoC from 2020 to 2025 (r=0.95, p=0.004) (Figure 4). 

**Figure 5:**
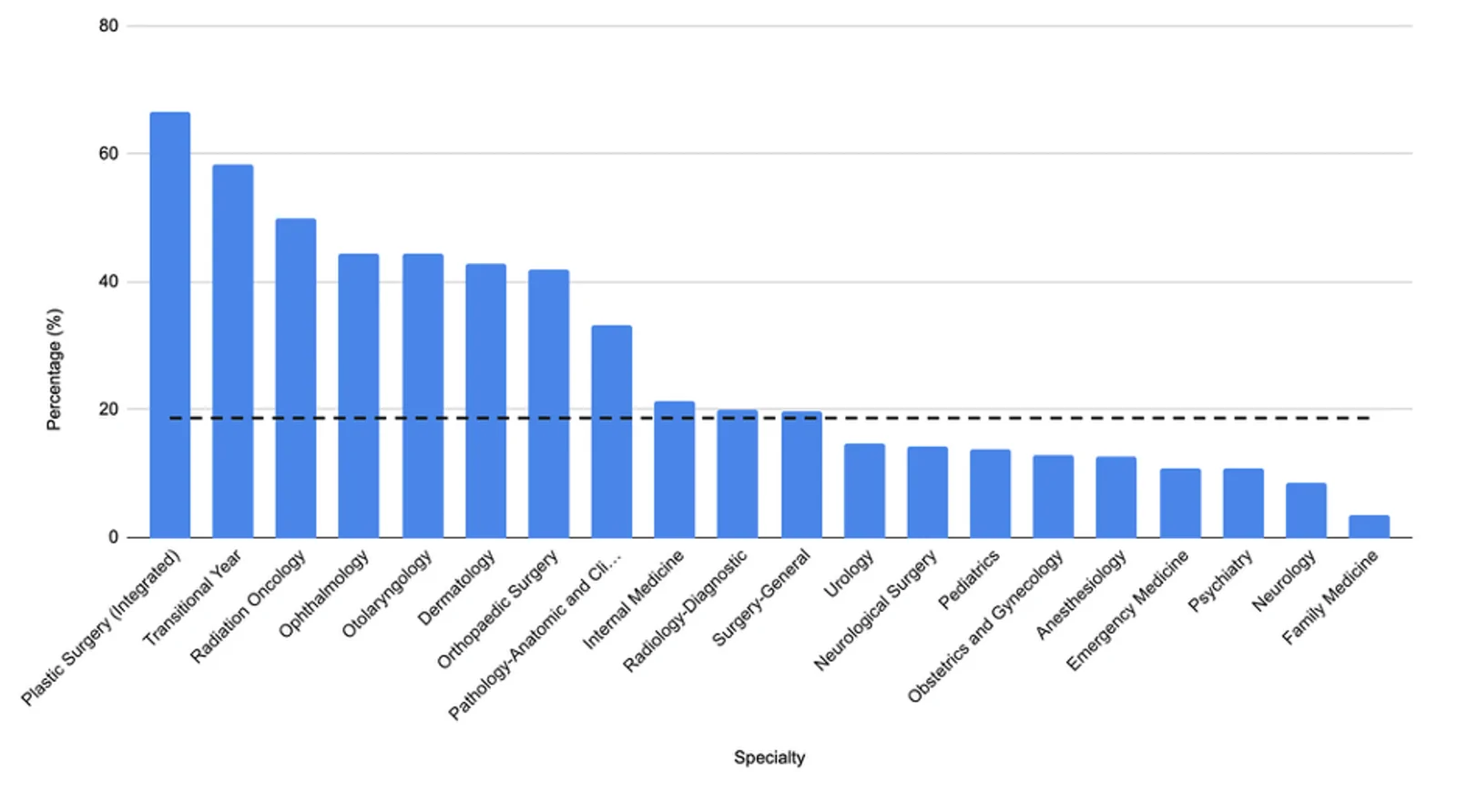
Percentage Of Each Specialty Completing the AoC Percentage of applicants matched to each specialty who completed the AoC (area of concentration). The blacked dash line represents the percentage of the class overall that matched and completed the AoC between 2022 and 2025.

We found that those who matched into plastic surgery, a highly competitive speciality, had the highest completion rate of the Biomedical Research AoC at 66.7%. The second and third highest Biomedical Research AoC completion rates were for those pursuing a transitional year (58.3%) and those who matched in radiation oncology (50%) (Figure 5). Students who matched into competitive medical specialities (M=31.96%, SD=6.45) had a significantly higher AoC completion rate than those applying to less competitive specialities (M=16.13%, SD=1.77), Welch’s t(4.60), p=4.62 × 10⁻⁵, d=2.92.

**Figure 6:**
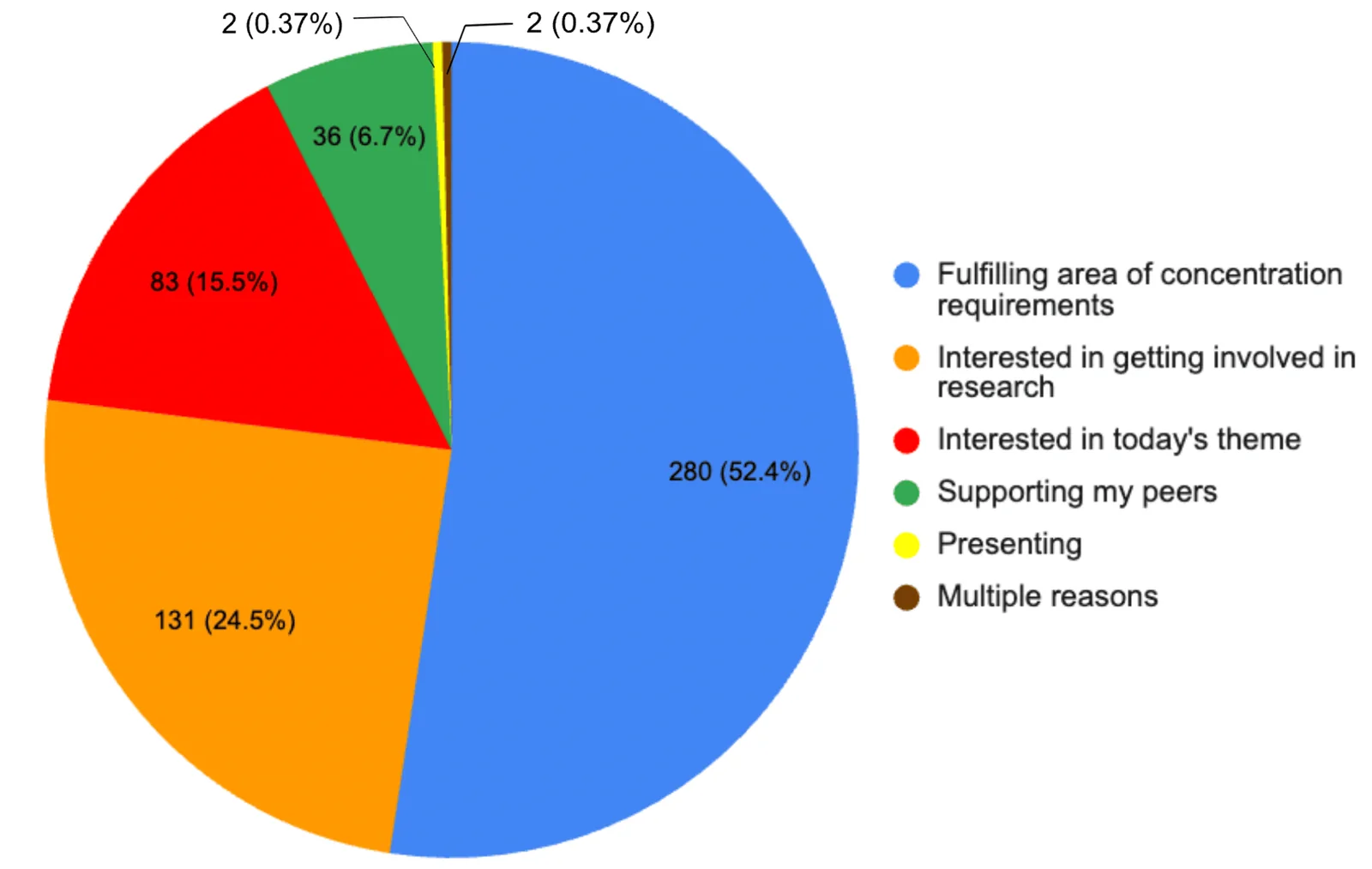
Reason for Attending Research Seminar Results of a survey question asking students their reason for attending the research seminar. Blue represents fulfilling areas of concentration requirements; orange represents interest in getting involved in research; red indicates interest in today’s theme; green indicates supporting their peers; yellow represents presenting; and brown indicates multiple reasons cited for attending.

In the survey of research seminar attendees distributed in the fall of 2025, we received a total of 534 responses from medical students. Of those surveyed, 96.8% (517) were first- and second-year medical students. Regarding student motivations for attending the research seminars, 52.4% (280) of attendees surveyed reported attending the research seminar to fulfil the Biomedical Research AoC requirements (Figure 6). The next most common reason for attending was interest in getting involved in research (131, 24.5%), followed by interest in today’s theme (83, 15.5%), supporting their peers (36, 6.7%), presenting (2, 0.37%) and multiple reasons (2, 0.37%) (Figure 6). Of the attendees surveyed, 97.2% (519) reported believing that attending the seminar series could help them identify research opportunities.

**Figure 7:**
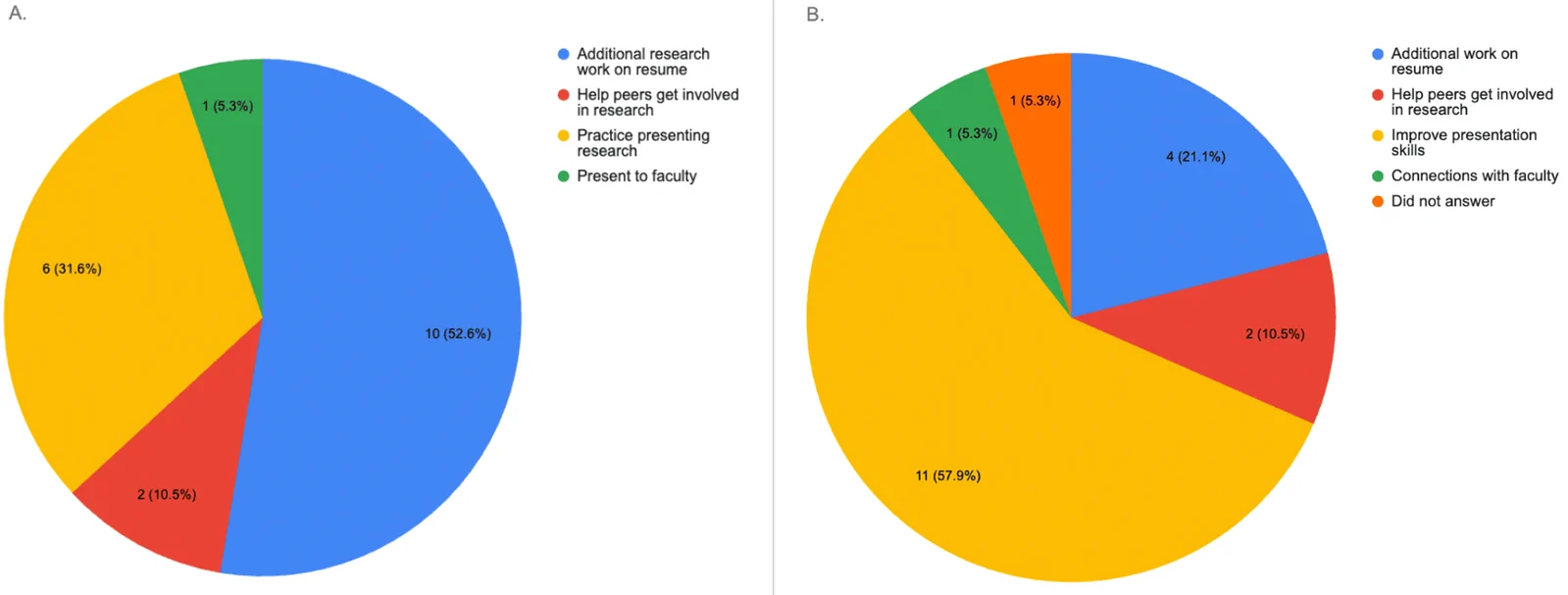
Motivation and Benefits of Presenting at Research Seminars. A. Pie chart depicting presenter’s motivation for presenting. Blue represents additional work on the resume, red represents helping peers get involved in research, yellow represents practising presenting research, and green represents presenting to faculty; B. Pie chart depicting presenter's perceived benefits of presenting at the research seminars. Yellow represents improving presentation skills, blue represents additional work on resume, red represents helping peers get involved in research, green represents connections with faculty and orange represents not answering the question.

As for the survey of research seminar presenters during the academic year 2025-2026, we received 19 responses out of 32 presenters, representing a response rate of 59.4%. Concerning motivations for presenting, the most common response was: additional research work on resume (52.6%, 10), followed by practice presentation skills (6, 31.6%), help peers get involved in research (2, 10.5%), and present to faculty (1, 5.3%) (Figure 7). When asked how they benefited from presenting, the most common response was to improve their presentation skills (11, 57.9%), followed by an additional work on resume (4, 21.1%), help peers get involved in research (2, 10.5%), build connections with faculty (1, 5.3%), and one did not respond (1, 5.3%) (Figure 7). The majority of presenters had previously presented at a research seminar (10, 52.6%). 

Presenters most commonly reported interests in pursuing the medical specialities of ophthalmology (4, 21.1%), orthopedic surgery (3, 15.8%), internal medicine (3, 15.8%), neurology (2, 10.5%), obstetrics and gynecology (2, 10.5%), and anesthesia (2, 10.5%). When asked about their comfort level when answering questions from their peers, the most common response was that they were not asked a question by their peers (8, 42.1%). When asked about answering questions from faculty, the most common responses were felt very comfortable (6, 31.6%) and felt a little comfortable (7, 36.8%). The full results of answers to these questions can be seen in Table 1. 

**Table 1:** Presenter Survey Results.

Survey Option	Number of Responses	Percentage of Responses (%)
Question: What specialty do you plan to pursue?		
Ophthalmology	4	21.1%
Orthopedic surgery	3	15.8%
Internal medicine	3	15.8%
Neurology	2	10.5%
Obstetrics and gynecology	2	10.5%
Anesthesia	2	10.5%
Urology	1	5.3%
Dermatology	1	5.3%
Not sure	1	5.3%
Question: How comfortable did you feel answering questions from other medical students?		
Very comfortable	5	26.3%
A little comfortable	3	15.8%
Indifferent	1	5.3%
A little uncomfortable	1	5.3%
Very uncomfortable	1	5.3%
Was not asked questions	8	42.1%
Question: How comfortable did you feel answering questions from faculty members/physicians?		
Very comfortable	6	31.6%
A little comfortable	7	36.8%
Indifferent	1	5.3%
A little uncomfortable	3	15.8%
Very uncomfortable	2	10.5%

## Discussion

Several findings from this study suggest that peer-led research seminars can serve as a valuable component of a medical school's Research Area of Concentration (AoC) or similar scholarly pathway. Over the study period, the number of students completing the Biomedical Research AoC increased significantly while attendance at student-led research seminars remained consistently high, demonstrating sustained student engagement with the program. Survey data further showed that fulfilling AoC requirements was the most common reason for attending research seminars, suggesting that participation in the seminar series was closely integrated with students' pursuit of structured scholarly training. Together, these findings may reflect growing student interest in formal research experiences, potentially driven by evolving expectations within the residency application process. This interpretation is supported by previous work demonstrating that medical students perceived research experiences to be increasingly important following the transition of USMLE Step 1 to pass/fail [15]. Similarly, a recent study found that the most common motivation for participating in extracurricular activities was to strengthen residency applications [16]. Collectively, these findings suggest that structured, peer-led research initiatives may provide an accessible avenue for students to develop scholarly skills while meeting the increasing emphasis on research engagement in residency selection.

Over half of the presenters surveyed in this study shared that their primary motivation for presenting at the research seminars was to bolster their resumes. This aligns with the previously discussed pressures for medical students to increase their scholarly productivity to match into competitive residency programs [3]. Yet, when asked how they benefited most from presenting at the seminar, over 60% of respondents shared that improvement of their research presentation skills was the primary benefit, and only 22.2% selected additional work on their resume as the primary benefit. This suggests that students may initially apply to the present to strengthen their resumes, but after presenting, their perception changes, and they recognize the ability to practice and improve their presentation skills as more valuable than the resume boost.

Not only does the Biomedical Research AoC provide a way for medical students to distinguish themselves by graduating with distinction in research, but it also provides a path to increase research productivity by presenting at the research seminars. In the match data from 2024 published by the National Resident Matching Program, the specialities that had the highest number of research works for matched applicants were neurological surgery (37.4), followed by plastic surgery (34.7), dermatology (27.7) and orthopedic surgery (23.8), which are amongst the most competitive specialities [9]. In our study, neurosurgery and orthopedic surgery were the two most common seminar themes. Our data indicate that those applying to competitive specialties utilize the peer-led research seminars and AoC to graduate with distinction in research, increase their number of research works, develop research skills, and learn about possible research opportunities from their peers. Additionally, those who matched into competitive specialities were more likely to have completed the Biomedical Research AoC than those matched into less competitive specialities. This makes sense, as a prior study interviewing program directors for residency programs found that program directors from the most competitive specialities were more likely to value research in their applicants [17]. Thus, programs such as the AoC may be especially beneficial for those applying to competitive subspecialties. 

Although the Biomedical Research AoC outlined in this study represents an institution-specific program, many medical schools have analogous opportunities including scholarly concentrations, research tracks, distinction pathways, and longitudinal research programs that could incorporate similar components. These programs often have longitudinal coursework that complements the medical school curriculum. However, our AoC additionally includes peer-led aspects such as the research seminars that function as a way for students to learn from their peers and gain additional research work [6,7]. As medical schools continue adapting to P/F assessment structures and evolving residency selection practices, understanding how these programs can benefit medical students and how their participation changes over time is increasingly valuable. For schools without formal research pathways, implementation of structured research programs may be worth considering, as these programs may provide students with opportunities for research involvement, mentorship, faculty networking, and scholarly skills development. For schools with similar programs, it may be beneficial to include peer-led components such as research seminars as a way for medical students to build their resume, improve presentation skills, and engage with their peers. 

Limitations

It is important to note the limitations of this study. The study was conducted at only one medical school. Many medical schools have different grading and ranking systems, making comparisons challenging. Other limitations include that we were only able to conduct the survey after USMLE Step 1 transitioned to P/F. If the survey had also been given before USMLE Step 1 transitioned to P/F, it would have greatly strengthened our findings and allowed us to directly compare how student attitudes have changed. Additionally, the small sample size of our presenters’ survey also limited our analysis and conclusions. It is important to note that the responses of the survey may not be representative of the entire time period of the study, and there may be bias in those responding, as those attending the research seminars likely attend and present due to perceived benefit. 

## Conclusions

In conclusion, this study provides important insights into how students can benefit from peer-led research seminars and formal research programs during medical school. Programs such as these are likely to become increasingly important following the transition of USMLE Step 1 to a pass/fail format and research becoming a more important component of matching to residency. Through the program described in this study, medical students are provided with the opportunity to get involved with research early in their medical school careers, with primarily M1s and M2s attending and presenting. These programs benefit both presenters and attendees, providing participants with important research presentation skills, connections with research opportunities, and additional research work for their residency applications, all of which are especially beneficial and utilized by those applying to competitive subspecialties for residency. 

## Appendices

Appendix A 

**Table 2:** Participation Survey for Research Seminar Attendees.

* Indicates required question
What is your full name (for attendance purposes)? *
ID: *
What year are you in medical school? *
M1
M2
M3
M4
Please choose your primary reason for joining the medical student research seminar series. *
Interested in getting involved in research
Interested in today's theme
Supporting my peers
Fulfilling area of concentration requirements
Other:
What medical specialty do you intend to pursue? *
Do you believe attending the seminar series may help you identify research opportunities? *
Yes
No

Appendix B 

**Table 3:** Survey for Presenters.

* Indicates required question
Please choose your motivation for presenting today. *
Practice presenting research
Additional research work on resume
Help peers get involved in research
Present to faculty
How do you feel you benefitted from presenting today?
Improve presentation skills
Connections with faculty
Help peers get involved in research
Additional research work on resume
How comfortable did you feel answering questions from other medical students?*
Very uncomfortable
A little uncomfortable
Indifferent
A little comfortable
Very comfortable
I was not asked questions by students
How comfortable did you feel answering questions from faculty members/physicians?*
Very uncomfortable
A little uncomfortable
Indifferent
A little comfortable
Very comfortable
I was not asked questions by faculty members/physicians
Have you presented before? *
Yes
No
If you answered yes to the above, how many times not including today?
Other:
What specialty do you plan to pursue? *
General Surgery
Orthopedic Surgery
Neurosurgery
Internal Medicine
Psychiatry
Family Medicine
OBGYN
Dermatology
Opthamology
Urology
Anesthesiology
Emergency Medicine
Interventional Radiology
Neurology
Otolaryngology
Pediatrics
Plastic Surgery
PMR
Radiology Oncology
Radiology - Diagnostic
Thoracic Surgery
Other:

## References

[1] Matthews CN, Estrada DC, George-Weinstein M, Claeson KM, Roberts MB (2019). Evaluating the influence of research on match success for osteopathic and allopathic applicants to residency programs. J Am Osteopath Assoc.

[2] Lin LO, Makhoul AT, Hackenberger PN (2020). Implications of pass/fail step 1 scoring: plastic surgery program director and applicant perspective. Plast Reconstr Surg Glob Open.

[3] Lichtbroun BJ, Rajagopalan A, Chua K (2024). How do I match? A survey study on the impact of step 1 becoming pass/fail. J Surg Educ.

[4] Hulou MM, Samaan CA, McLouth CJ (2023). Competitive neurosurgery residency programs: predictors of matching outcome and research productivity. Clin Neurol Neurosurg.

[5] Fan RR, Aziz F, Wittgen CM, Williams MS, Smeds MR (2023). A survey of vascular surgery program directors: perspectives following USMLE Step 1 conversion to pass/fail and virtual only interviews. Ann Vasc Surg.

[6] Stebbins S, Sanders JL, Vukotich CJ Jr, Mahoney JF (2011). Public health area of concentration: a model for integration into medical school curricula. Am J Prev Med.

[7] Alkhatib HH, Beach MC, Gebo KA (2023). The association of a scholarly concentrations program with medical students' matched residencies. Med Educ Online.

[8] (2026). Areas of concentration. https://www.nymc.edu/som/education/md/areas-of-concentration/.

[9] National Resident Matching Program (2024). Charting Outcomes™: Characteristics of U.S. MD Seniors Who Matched to Their Preferred Speciality: 2024 Main Residency Match. Medical Schools.

[10] Harris CR, Millman KJ, van der Walt SJ (2020). Array programming with NumPy. Nature.

[11] Virtanen P, Gommers R, Oliphant TE (2020). SciPy 1.0: fundamental algorithms for scientific computing in Python. Nat Methods.

[12] (2026). Python. Python Software Foundation. Published online.

[13] Hunter JD (2007). Matplotlib: A 2D graphics environment. Comput Sci Eng.

[14] Waskom M (2021). Seaborn: statistical data visualization. J Open Source Softw.

[15] Currie M, Hammond C, Martinez OP, Lane-Cordova A, Cook J (2024). The impact of United States Medical Licensing Examination step 1 transitioning to pass/fail on medical student perception of research needed to match into one’s preferred specialty. Cureus.

[16] Almasry M, Kayali Z, Alsaad R, Alhayaza G, Ahmad MS, Obeidat A, Abu-Zaid A (2017). Perceptions of preclinical medical students towards extracurricular activities. Int J Med Educ.

[17] Wolfson RK, Fairchild PC, Bahner I (2023). Residency program directors’ views on research conducted during medical school: a national survey. Acad Med.

